# Supplementary material for: Influenza Vaccination Uptake and Hesitancy among Healthcare Workers in Early 2021 at the Start of the COVID-19 Vaccine Rollout in Cape Town, South Africa
Source: Vaccines (Basel). 2022 Jul 25;10(8):1176. doi: 10.3390/vaccines10081176 (PMC9332543; doi:10.3390/vaccines10081176)
Supplement: Supplementary file 1 [file vaccines-10-01176-s001.zip › vaccines-1781364-supplementary.pdf]

Supplementary Table S1: Questionnaire

Participants ID ……… [number to be assigned by study investigators].

Initials…………………………………………………………………………..

Date of birth……………………………………………………………………….

Healthcare worker role:

Doctor, Nurse, Nursing assistant, Counsellor, Radiologist, Audiologist, Occupational therapist, Physiotherapist, Clinical research worker, Pharmacist, Admin Support staff, Other (specify………………..)

Gender:

1=Female, 2=Male, 3=Other

Education attainment:

Matric, Diploma, BSc, Honours, MBBS/MBChB, MSc, PhD, Other (Specify…………….)

Personal income:

Less than R10000 per month, R10000-R50000 per month, More than R50000 per month

Religion:……………………………………………………………………………….

Date……………………………………………………………………………………….

Instruction: Please evaluate how much you disagree or agree with the following statements

A. Influenza vaccination

1.	I have received at least one dose of the influenza vaccine in the past	1= No, 2= Yes
2.	During the next influenza season, I will take the influenza vaccine.	1 = strongly disagree, 2 = moderately disagree, 3 = slightly disagree, 4 = neutral, 5 = slightly agree, 6 = moderately agree, 7 = strongly agree
3.	Influenza vaccination is compatible with my religion	1 = strongly disagree, 2 = moderately disagree, 3 = slightly disagree, 4 = neutral, 5 = slightly agree, 6 = moderately agree, 7 = strongly agree
4.	I am completely confident that influenza vaccines are safe.	1 = strongly disagree, 2 = moderately disagree, 3 = slightly disagree, 4 = neutral, 5 = slightly agree, 6 = moderately agree, 7 = strongly agree
5.	Influenza vaccinations are effective.	1 = strongly disagree, 2 = moderately disagree, 3 = slightly disagree, 4 = neutral, 5 = slightly agree, 6 = moderately agree, 7 = strongly agree
6.	Regarding influenza vaccines, I am confident that public authorities decide in the best interest of the community.	1 = strongly disagree, 2 = moderately disagree, 3 = slightly disagree, 4 = neutral, 5 = slightly agree, 6 = moderately agree, 7 = strongly agree

7.	Influenza vaccination is unnecessary because influenza is not common anymore.	1 = strongly disagree, 2 = moderately disagree, 3 = slightly disagree, 4 = neutral, 5 = slightly agree, 6 = moderately agree, 7 = strongly agree
8.	My immune system is so strong, it also protects me against influenza	1 = strongly disagree, 2 = moderately disagree, 3 = slightly disagree, 4 = neutral, 5 = slightly agree, 6 = moderately agree, 7 = strongly agree
9.	Influenza is not so severe that I should get vaccinated.	1 = strongly disagree, 2 = moderately disagree, 3 = slightly disagree, 4 = neutral, 5 = slightly agree, 6 = moderately agree, 7 = strongly agree
10.	Everyday stress prevents me from getting the influenza vaccine	1 = strongly disagree, 2 = moderately disagree, 3 = slightly disagree, 4 = neutral, 5 = slightly agree, 6 = moderately agree, 7 = strongly agree
11.	For me, it is inconvenient to receive influenza vaccinations	1 = strongly disagree, 2 = moderately disagree, 3 = slightly disagree, 4 = neutral, 5 = slightly agree, 6 = moderately agree, 7 = strongly agree
12.	Visiting a vaccination clinic makes me feel uncomfortable; this keeps me from getting vaccinated against influenza	1 = strongly disagree, 2 = moderately disagree, 3 = slightly disagree, 4 = neutral, 5 = slightly agree, 6 = moderately agree, 7 = strongly agree
13.	When I think about getting vaccinated against influenza, I weigh benefits and risks to make the best decision possible.	1 = strongly disagree, 2 = moderately disagree, 3 = slightly disagree, 4 = neutral, 5 = slightly agree, 6 = moderately agree, 7 = strongly agree
14.	For every influenza vaccine dose, I closely consider whether it is useful for me.	1 = strongly disagree, 2 = moderately disagree, 3 = slightly disagree, 4 = neutral, 5 = slightly agree, 6 = moderately agree, 7 = strongly agree
15.	It is important for me to fully understand the topic of vaccination, before I get vaccinated against influenza.	1 = strongly disagree, 2 = moderately disagree, 3 = slightly disagree, 4 = neutral, 5 = slightly agree, 6 = moderately agree, 7 = strongly agree
16.	When everyone is vaccinated against influenza, I don't have to get vaccinated, too.	1 = strongly disagree, 2 = moderately disagree, 3 = slightly disagree, 4 = neutral, 5 = slightly agree, 6 = moderately agree, 7 = strongly agree
17.	I get vaccinated against influenza because I can also protect people with a weaker immune system.	1 = strongly disagree, 2 = moderately disagree, 3 = slightly disagree, 4 = neutral, 5 = slightly agree, 6 = moderately agree, 7 = strongly agree
18.	Vaccination is a collective action to prevent the spread of diseases like influenza.	1 = strongly disagree, 2 = moderately disagree, 3 = slightly disagree, 4 = neutral, 5 = slightly agree, 6 = moderately agree, 7 = strongly agree
**B. Covid-19 vaccination**		
19.	I have received a COVID-19 vaccine	1= No, 2= Yes
21.	I will take the COVID-19 vaccine when one becomes available	1 = strongly disagree, 2 = moderately disagree, 3 = slightly disagree, 4 = neutral, 5 = slightly agree, 6 = moderately agree, 7 = strongly agree
21	COVID-19 vaccination is compatible with my religion	1 = strongly disagree, 2 = moderately disagree, 3 = slightly disagree, 4 = neutral, 5 = slightly agree, 6 = moderately agree, 7 = strongly agree

22.	I am completely confident that Covid-19 vaccines are safe.	1 = strongly disagree, 2 = moderately disagree, 3 = slightly disagree, 4 = neutral, 5 = slightly agree, 6 = moderately agree, 7 = strongly agree
23.	Covid-19 vaccination will be effective.	1 = strongly disagree, 2 = moderately disagree, 3 = slightly disagree, 4 = neutral, 5 = slightly agree, 6 = moderately agree, 7 = strongly agree
24.	Regarding Covid-19 vaccines, I am confident that public authorities decide in the best interest of the community.	1 = strongly disagree, 2 = moderately disagree, 3 = slightly disagree, 4 = neutral, 5 = slightly agree, 6 = moderately agree, 7 = strongly agree
25.	Covid-19 vaccination is unnecessary because Covid-19 is not common anymore.	1 = strongly disagree, 2 = moderately disagree, 3 = slightly disagree, 4 = neutral, 5 = slightly agree, 6 = moderately agree, 7 = strongly agree
27.	My immune system is so strong, it also protects me against Covid-19	1 = strongly disagree, 2 = moderately disagree, 3 = slightly disagree, 4 = neutral, 5 = slightly agree, 6 = moderately agree, 7 = strongly agree
28.	Covid-19 infection is not so severe that I should get vaccinated.	1 = strongly disagree, 2 = moderately disagree, 3 = slightly disagree, 4 = neutral, 5 = slightly agree, 6 = moderately agree, 7 = strongly agree
29.	Everyday stress will prevent me from getting vaccinated against Covid-19	1 = strongly disagree, 2 = moderately disagree, 3 = slightly disagree, 4 = neutral, 5 = slightly agree, 6 = moderately agree, 7 = strongly agree
30.	For me, it is inconvenient to receive vaccinations against Covid-19.	1 = strongly disagree, 2 = moderately disagree, 3 = slightly disagree, 4 = neutral, 5 = slightly agree, 6 = moderately agree, 7 = strongly agree
31.	Visiting the vaccination clinic will make me feel uncomfortable; this will keep me from getting vaccinated against Covid-19.	1 = strongly disagree, 2 = moderately disagree, 3 = slightly disagree, 4 = neutral, 5 = slightly agree, 6 = moderately agree, 7 = strongly agree
32.	When I think about getting vaccinated against Covid-19, I weigh benefits and risks to make the best decision possible.	1 = strongly disagree, 2 = moderately disagree, 3 = slightly disagree, 4 = neutral, 5 = slightly agree, 6 = moderately agree, 7 = strongly agree
33.	For every Covid-19 vaccine dose, I will closely consider whether it is useful for me.	1 = strongly disagree, 2 = moderately disagree, 3 = slightly disagree, 4 = neutral, 5 = slightly agree, 6 = moderately agree, 7 = strongly agree
34.	It is important for me to fully understand the topic of vaccination, before I get vaccinated against Covid-19.	1 = strongly disagree, 2 = moderately disagree, 3 = slightly disagree, 4 = neutral, 5 = slightly agree, 6 = moderately agree, 7 = strongly agree
35.	When everyone is vaccinated against Covid-19, I don't have to get vaccinated, too.	1 = strongly disagree, 2 = moderately disagree, 3 = slightly disagree, 4 = neutral, 5 = slightly agree, 6 = moderately agree, 7 = strongly agree
36.	I will get vaccinated against Covid-19 because I can also protect people with a weaker immune system.	1 = strongly disagree, 2 = moderately disagree, 3 = slightly disagree, 4 = neutral, 5 = slightly agree, 6 = moderately agree, 7 = strongly agree
37.	Vaccination is a collective action to prevent the spread of diseases like Covid-19.	1 = strongly disagree, 2 = moderately disagree, 3 = slightly disagree, 4 = neutral, 5 = slightly agree, 6 = moderately agree, 7 = strongly agree
